# The cumulative bridge: how long-term physical activity and social engagement gradually enhance sleep health in aging adults

**DOI:** 10.1186/s12889-026-27502-1

**Published:** 2026-04-30

**Authors:** Guiping Zhao, Ketao Zhang, Xiaotian Li

**Affiliations:** Capital University of Physical Education, No.11, Beisanhuan West Road, Haidian District, Beijing, China

**Keywords:** Physical activity, Social activity, Sleep duration, Middle-aged and older adults, Ageing population

## Abstract

**Background and objectives:**

Sleep disturbances are significant public health issues for middle-aged and older adults. While cross-sectional research shows associations, a comprehensive understanding of the long-term, cumulative dynamics among multiple health behaviours and sleep duration remains underdeveloped. This study examines the distinct temporal effects and synergistic potential of sustained physical activity and social engagement on sleep duration in the ageing population.

**Research methodology:**

Using five waves of longitudinal panel data from the China Health and Retirement Longitudinal Study (CHARLS, 2011–2018), we analysed 5,082 participants (57,716 observations). We employed a nested fixed-effects model with interactions between behaviours and time (survey wave) to control for individual unobservable heterogeneity and capture dynamic effects. Control variables included marital status, gender, age, education level, drinking habits, and chronic diseases.

**Results:**

The direct effect of physical activity on sleep duration was negative in the short term. Crucially, its interaction with time showed a significant positive cumulative effect. Social activity also demonstrated a positive temporal effect, though the magnitude was notably smaller. Marital status exhibited a significant protective effect, and drinking habits were significantly negative. The temporal beneficial impact of physical activity was most pronounced in the middle school education group.

**Conclusions​:**

Long-term engagement in both physical activity and social activities positively enhances sleep duration, with physical activity having a more substantial and long-lasting protective impact that accrues over time. These findings underscore the need for public health policies to emphasise sustained, long-term interventions and consider education-level heterogeneity to maximise sleep benefits for ageing adults.

## Introduction

With the acceleration of population ageing, the health problems of middle-aged and older adults are receiving increasing attention, among which sleep problems are particularly prominent. Sleep is a crucial foundation for maintaining physical and mental health, as it impacts individuals’ physiological functions, psychological state, and cognitive abilities [1]. In recent years, the quality of sleep in the middle-aged and older adult population has shown a declining trend globally, with a significant increase in sleep deprivation and sleep disorders, which seriously affects their quality of life and health [2]. Despite extensive research, a considerable gap remains in understanding the long-term, dynamic, and cumulative effects of health behaviours on sleep duration. Most existing studies, often relying on cross-sectional data, are limited in their ability to capture these temporal trends and causal relationships. Research shows that the average sleep duration in China’s middle-aged and elderly population is decreasing yearly, and the subjective quality of sleep—which refers to individuals’ personal assessment and satisfaction with their sleep experience, including perceived sleep adequacy, refreshment upon waking, and overall sleep satisfaction—continues to decline [3, 4]. This phenomenon poses a threat to individual health. It may also exacerbate the burden of medical care on society, making it a public health problem that requires urgent attention.

Existing studies have shown that the factors affecting sleep duration in middle-aged and older adults are complex and diverse, including physiological, psychological, and social factors [5, 6]. This study explores how specific health behaviours—namely marital status, alcohol consumption patterns, physical exercise participation, and social activity engagement—affect sleep duration in middle-aged and older adults. To address these critical gaps, this study systematically investigates the long-term, cumulative effects of key health behaviours, specifically physical activity and social engagement, on sleep duration in ageing adults. The following core questions guide our research: (1) What is the long-term, dynamic relationship between sustained physical activity and social engagement, and sleep duration in middle-aged and older adults? (2) How do physical activity and social activity interact to produce synergistic effects on long-term sleep health? Through systematic analyses of these questions, this study aims to provide a theoretical basis and practical guidance for improving sleep duration among middle-aged and older adults.

To provide a robust theoretical grounding for these inquiries, this study draws upon Life Course Theory and the Cumulative Advantage/Disadvantage (CAD) hypothesis. Life Course Theory posits that health trajectories are shaped by long-term exposure to behavioural patterns rather than isolated events [7]. Within this framework, physical activity and social engagement are viewed not merely as immediate health behaviours but as cumulative capital that builds physiological and psychosocial resilience over time. The CAD hypothesis further suggests that early and sustained adoption of these behaviours can widen health disparities—such as sleep health—between active and inactive individuals as they age [8]. By integrating these theoretical perspectives, we propose the “Cumulative Bridge” framework to explain how the benefits of sustained activity accrue to bridge the gap between ageing and declining sleep health [9].

Previous research has established that health behaviours, such as marital status, drinking behaviour, physical activity, and social activities, are closely related to sleep duration among middle-aged and older adults. Marital status is an important social factor that affects the sleep of middle-aged and older adults. Studies have found that the advantages of being married in terms of emotional support and life stability can significantly improve their sleep duration, while unmarried, divorced, or widowed people are more likely to have sleep problems due to a lack of emotional support [10, 11]. The quality of the marital relationship can also significantly impact sleep outcomes, with high-quality marital relationships considerably reducing the incidence of sleep disorders. In contrast, marital conflict can harm sleep [12].

The negative impact of unhealthy lifestyles (e.g., drinking behaviours) on the quality of sleep in middle-aged and older adults should not be overlooked. Although alcohol consumption may help with sleep in the short term, long-term consumption of alcohol can lead to fragmented sleep, reduced deep sleep, and early awakenings, which can significantly reduce the quality of sleep [13]. Limiting drinking behaviour and promoting healthy lifestyles are thus essential measures to improve sleep problems in middle-aged and older people.

The impact of health behaviours on sleep has attracted increasing attention in recent years. As an effective way to improve sleep, the long-term effect of has been widely recognised. Regular low- and medium-intensity exercise can regulate physiological rhythms, alleviate psychological pressure, and improve sleep duration by enhancing body functions. In contrast, short-term high-intensity exercise may lead to fatigue or post-exercise euphoria, which may impair sleep [14–16]. Socialisation, as an essential form of social support, has also been shown to indirectly enhance sleep duration by reducing loneliness and improving mental health [17]. Studies have shown that middle-aged and older adults who are socially active have significantly better sleep duration and quality than those who are socially inactive [18].

Many studies have examined the effects of health behaviours on sleep. However, the following shortcomings still exist: (1) most of the studies mainly focus on the effects of single health behaviors (e.g., physical activity or alcohol consumption behaviors) on sleep and lack a comprehensive analysis of multiple health behaviors; (2) there are fewer studies on the long-term dynamic effects of health behaviors on sleep and their interactions; and (3) most of the existing studies are based on cross-sectional data, which cannot establish causal relationships between variables. Based on the five-wave panel data from the China Health and Retirement Longitudinal Study (CHARLS), this study uses nested model analysis to systematically investigate the long-term dynamic effects of various health behaviours, specifically examining the long-term and interactive effects of physical and social activity on sleep duration.

Based on the five-wave panel data of the China Health and Retirement Longitudinal Study (CHARLS), this study uses nested model analysis to systematically investigate the long-term dynamic effects of health behaviours such as marital status, alcohol consumption, physical activity, and social activity on sleep duration and their interactive effects. This study aims to provide unique empirical evidence on how health behaviours influence sleep duration through longitudinal mechanisms, specifically quantifying the temporal distinct impact of both physical and social activities. Moreover, this research provides a crucial practical reference for developing evidence-based interventions to improve sleep outcomes among middle-aged and elderly populations, highlighting the cumulative benefits of sustained engagement and the potential synergistic effects between these two key behaviours.

**Fig. 1:**
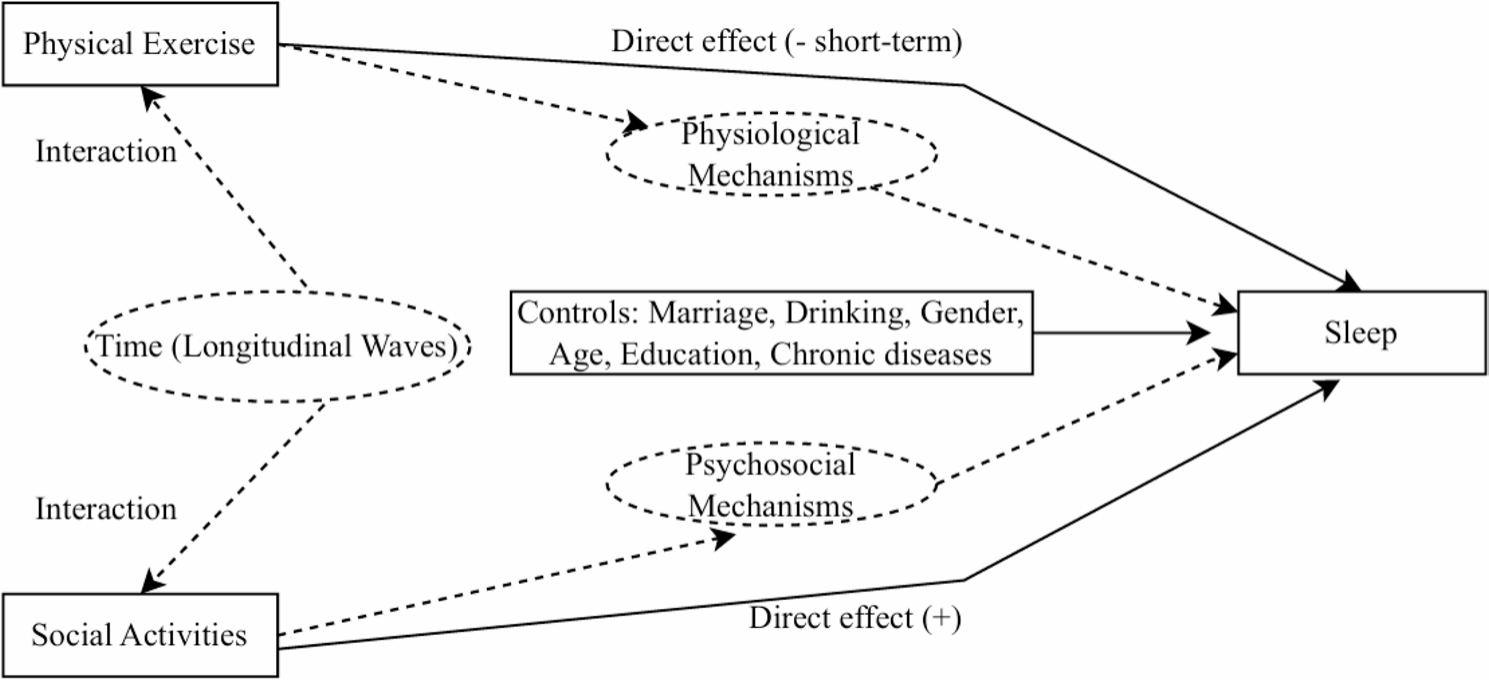
Research framework diagram

Figure 1 presents the conceptual framework of this study. exercise, physical activity, and social activities are the key independent variables that affect sleep duration among middle-aged and older adults through both direct effects and interactions with time. Marital status, drinking, gender, age, education, and chronic diseases are included as control variables. Two potential mechanisms are proposed: (1) physiological mechanisms, where exercise improves circadian rhythms and physical functions, thereby enhancing sleep over time; and (2) psychosocial mechanisms, where social participation reduces loneliness and improves emotional well-being, thereby promoting better sleep.

## Methods of analysis

### Data sources

This paper uses data from the China Health and Retirement Longitudinal Study (CHARLS). CHARLS is a representative longitudinal survey of people aged 45 and above in mainland China, conducted by the National Development Research Institute of Peking University, covering 150 counties/districts, 17,708 households, and more than 33,600 interviewees aged 45 and above, and collecting multi-dimensional information on socio-economic and health conditions to meet the needs of scientific research on ageing. Its national baseline survey was conducted in 2011-12, followed by four rounds of follow-up surveys using conventional questionnaires in 2013, 2015, 2018, and 2020, and the Life Course Survey of Middle-aged and Older Adults in China was completed in 2014. In late 2019 and early 2020, the COVID-19 pandemic broke out in China. To promptly document the impact of the COVID-19 pandemic on the lives and health of middle-aged and older adults in China, additional information related to the pandemic was collected in the 5th round of the survey in 2020. All participants gave written informed consent during the original CHARLS surveys; the dataset provided to researchers is fully de-identified.

The data used in this paper are a panel dataset formed by manually merging the five waves from 2011 to 2020, providing an excellent source for exploring the relationship between physical activity and sleep duration. The panel data structure was first established during data cleaning, followed by a balance test to ensure the dataset met the requirements for panel data. The variable-cleaning process recoded the education level to generate a new variable; the sleep duration variable was truncated to reduce the impact of extreme values on analysis results. Social activity variables were merged and processed to generate new variables and recoded by social activity frequency. The age variable was set to a minimum of 45 years, and a squared term for age was included to analyse non-linear effects. To ensure data integrity, observations with missing values were excluded, leaving the remaining data non-missing and usable for subsequent analyses.

### Analytical sample selection

**Fig. 2:**
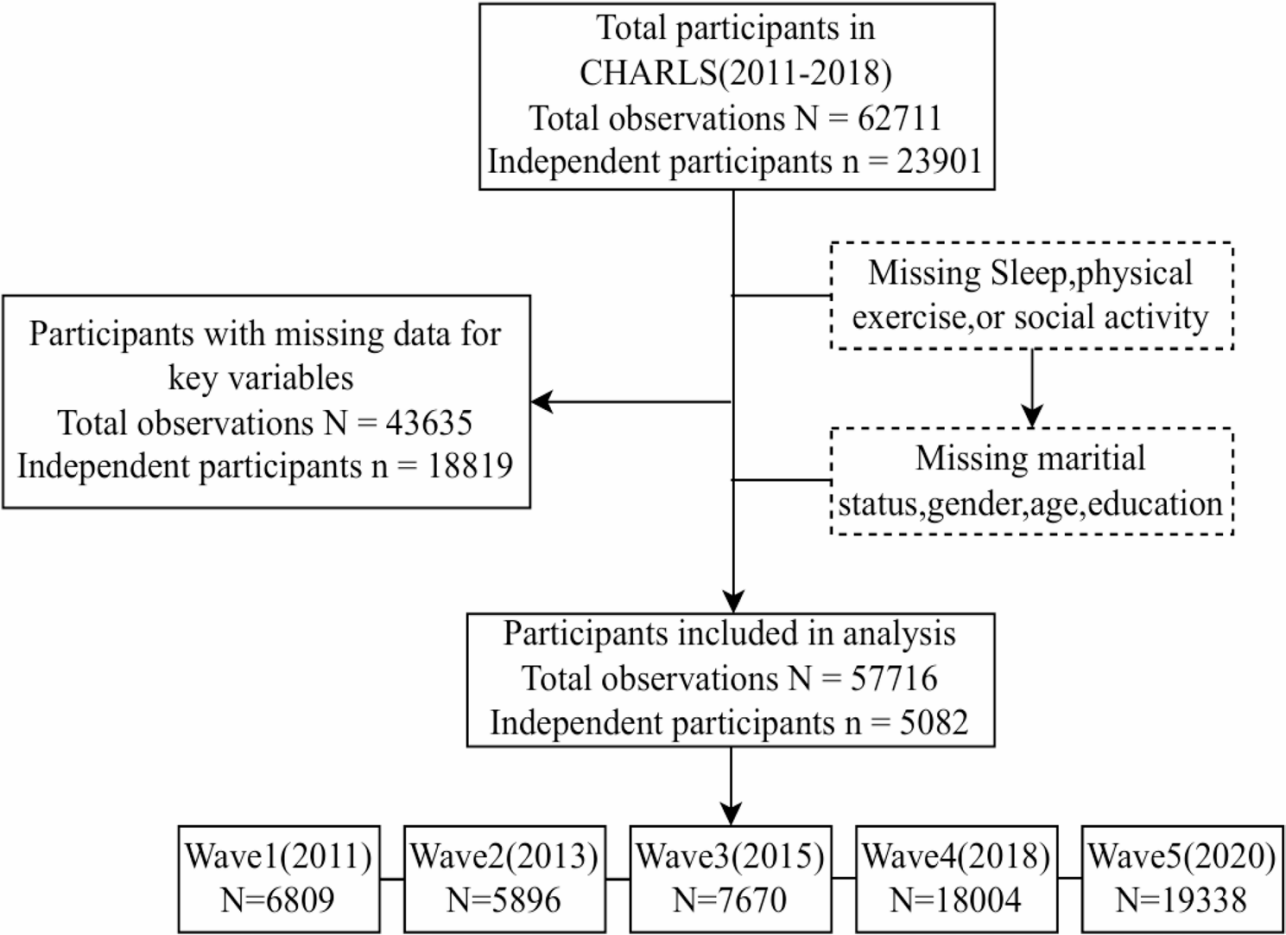
Flowchart of participant selection

To ensure the rigour of our analysis, we performed a strict data screening process, as detailed in Fig. 2. From an initial dataset of 23,901 independent participants and 62,711 observations, we applied the listwise deletion requirement of the fixed-effects regression model. This involved excluding all observations with missing values in our primary independent and dependent variables, as well as essential control variables, including sleep, marital status, age, urban/rural residence, education, drinking, chronic diseases, social activity, and physical activity. Our final analytical cohort consisted of 5,082 independent participants and 57,716 observations.

### Variant

The core independent variables were physical activity and social engagement. In this study, we categorised physical activity based on whether respondents engaged in regular physical activity in their daily lives. While we acknowledge that a binary indicator (participation vs. non-participation) limits the assessment of dose-response relationships and intensity, this specification was chosen to minimise recall bias and measurement error, which are prevalent in self-reported intensity data among older adults. By focusing on regular engagement, we capture the ‘habit formation’ aspect of behaviour, which is critical for long-term health accumulation, rather than episodic intensity. The variable is a binary indicator (0 = no participation, 1 = participation) based on participants’ responses to questions about their engagement in structured physical activities, such as walking, jogging, swimming, tai chi, or other forms of exercise. The measurement relies on participants’ self-assessment of their physical activity engagement without specific quantitative thresholds for intensity or duration. Without specific quantitative thresholds for intensity or duration. While CHARLS provides data to calculate metabolic equivalents (METs), we opted for a binary specification (participation vs. non-participation) for this longitudinal analysis. Given the advanced age of the cohort, self-reported duration and intensity are frequently subject to significant recall bias and measurement error. A binary indicator provides a more robust and reliable measure of behavioural “habit formation” and adherence over the nine years, minimising the noise associated with precise intensity estimation in an ageing population. Sleep duration, the dependent variable, is derived from question DA049 in the questionnaire: “In the past month, on average, how many hours did you fall asleep each night?” The sleep time was truncated to a minimum of 1 hour and a maximum of 10 hours.

Control variables included gender, marital status, age, age squared, education level, drinking habits, chronic disease status, interaction terms between social activity and survey wave, and interaction terms between physical activity and survey wave. Gender was coded as 1 for men and 2 for women; marital status was coded as 1 for married individuals and 2 for unmarried, divorced, or widowed individuals. The hukou variable was coded as 1 for urban and 2 for rural residents. Age was treated as a continuous variable, with individuals younger than 45 grouped as 45 years old for data cleaning, grouping, and outlier treatment purposes. To capture potential non-linear relationships between age and sleep duration, a quadratic age term (age²) was included in the model specification. This specification was predetermined based on theoretical expectations that sleep duration may follow a curvilinear pattern with age, initially remaining stable in middle age before declining more rapidly in later years. The quadratic specification allows for the identification of turning points in the age-sleep duration relationship. Education level was categorised as 1 for primary school and below, 2 for middle school, and 3 for high school or above. Drinking habits were coded as 0 for non-drinkers and 1 for drinkers; chronic disease status was coded as 0 for those without chronic disease and 1 for those with chronic disease. Participation in social activities was coded as 0 for non-participants and 1 for participants. The time variable in this study is operationalised as discrete survey waves, coded as 1, 2, 3, 4, and 5 corresponding to the 2011, 2013, 2015, 2018, and 2020 CHARLS data collection periods, respectively. The interaction terms between physical activity and time, as well as between social activity and time, capture how the effects of these behaviours on sleep duration change over the approximately 9-year study period. This discrete-time specification allows for the examination of nonlinear temporal effects while maintaining the interpretability of coefficients.

The selection of control variables was based on established literature and theoretical considerations. Marital status was included as it provides emotional and social support that may influence sleep patterns [19]. Gender and age were controlled for due to documented physiological differences in sleep patterns across demographic groups. Education level serves as a proxy for socioeconomic status and health literacy, which may affect sleep hygiene practices. Drinking habits were included as alcohol consumption is known to disrupt sleep architecture. Chronic disease status was controlled for, as medical conditions directly impact sleep duration. While other potential confounders, such as specific mental health conditions, medication use, and detailed socioeconomic indicators beyond education, may influence sleep duration, the current analysis focuses on the primary behavioural factors while acknowledging these limitations.

Variables were defaulted to the first category as the reference group, except for marital status, for which the unmarried category was used. Observations with missing values were excluded to ensure the dataset was complete (see Table 1 for details).

### Research methodology

In this paper, we use data from the China Health and Retirement Longitudinal Study (CHARLS) to examine the effects of physical activity, social activity, marital status, gender, age, and other factors on sleep duration among middle-aged and elderly individuals, using a fixed-effects panel-data model with nested interaction terms. The “nested” structure refers to the hierarchical inclusion of interaction terms between key variables (physical activity, social activity) and time within the main fixed-effects framework, allowing for the examination of how treatment effects vary across survey waves. The nested model effectively controls for unobserved individual heterogeneity, yielding more accurate estimates. The specific model is structured as follows:

1 $$Y_{it}=\beta_{1}X_{it}1+\beta_{2}X_{it}2+\cdot\cdot\cdot+\beta_{k}X_{it}k+{u}_{i}+\varepsilon_{it}$$

Where $\:{Y}_{it}$ is the dependent variable for an individual $\:i$ at time $\:t$, indicating sleep duration?. $\:{X}_{it}$ Represents the independent variables of the individual at time $\:t$, including marital status, gender, age, age squared, education level, drinking habits, chronic diseases, interaction term between participation in social activities and survey wave, and interaction term between physical activity and survey wave; $\:{u}_{i}$ represents individual fixed effects, controlling for individual unobservable heterogeneity, and $\:{\varepsilon\:}_{it}$ is a random error term. The respective variables’ influence coefficients on sleep duration can be obtained by estimating the above model, where Beta represents the coefficients. The analysis was conducted using Stata 18 software.

In the process of model estimation, this paper focuses on the following aspects: (1) exploring the effect of marriage on the sleep duration of middle-aged and older adults people by comparing married and unmarried groups; (2) examining the effect of the interaction of gender with other variables (e.g., physical activity) on sleep duration; (3) analyzing the long-term effects of physical activity and its interaction with time on sleep duration. Control variables included age, age squared, education level, drinking habits, and chronic disease status to reduce potential confounding bias; the model also introduced a time variable and its interaction term with other independent variables to capture the dynamic effects of temporal changes on sleep duration.

The estimation results from the nested model provide a clearer understanding of how marital status, gender, physical activity, and other factors influence sleep duration in middle-aged and older adults. These findings contribute to the theoretical framework and offer policy recommendations aimed at addressing the sleep issues faced by this demographic.

### Ethics statement

Ethics approval for this study was not separately required because the analysis was conducted on secondary, de-identified data from the China Health and Retirement Longitudinal Study (CHARLS). CHARLS was approved by the Biomedical Ethics Review Committee of Peking University (IRB00001052-11015), and all participants provided written informed consent at enrollment. No additional consent was needed for this secondary analysis.

All procedures performed in studies involving human participants were in accordance with the ethical standards of the institutional and/or national research committee and with the 1964 Helsinki declaration and its later amendments or comparable ethical standards.

## Results

### Descriptive statistical analysis

The descriptive statistical analysis results in Table 1 show that the average sleep duration of the middle-aged and older adults in the sample was close to 6 h (SD = 1.922). The proportion of the sample that did not participate in physical activity was 10.8%, and the proportion of those who did participate in physical activity was 89.2%. The percentage of married individuals in the marital status variable was 85.5%, while the percentage of unmarried individuals was 14.5%. In the gender variable, the mean value was 1.468 (SD = 0.499), with 1 representing males and 2 representing females, indicating that the proportion of men is 53.2% and the proportion of women is 46.8%. In the education variable, 48.8% had a lower secondary education, and 51.2% had a higher secondary education. The proportion of urban households is 23.5%, and the proportion of rural households is 76.5%. The percentage of those who did not drink alcohol was 65.8%, and the percentage of those who did consume alcohol was 34.2%. In the variable for chronic disease, 78.1% of the respondents had no chronic disease, and 21.9% had a chronic disease. The percentage of those who did not participate in social activities was 55.7%, and the percentage of those who did participate in social activities was 44.3%.

**Table 1:** Descriptive Statistics of Demographic Characteristics and Key Variables (*N* = 5,082)

Variable type	variable name	Variable Definition	average value	standard deviation
implicit variable	sleep duration	Continuity variables;	6.170	1.922
control variable	marital status	Married = 1; unmarried = 2;	1.145	0.352
	gender	Male = 1; Female = 2;	1.468	0.499
	age	Continuity variables;	61.910	10.010
	academic qualifications	Primary schools and below = 1; Junior high school = 2; Senior high school and above = 3;	1.465	0.707
	Square Subdivision of Age	Continuity variables;	3933	1293
	population	Urban = 1; rural = 2;	1.766	0.423
	Have you been drinking alcohol?	No = 0; Yes = 1;	0.342	0.474
	Presence of chronic diseases	No = 0; Yes = 1;	0.781	0.414
	Participation in social activities	No participation = 0; participation = 1;	0.443	0.497
	Wave	Continuity variables;	3.644	1.346
independent variable	Exercise or not	No = 0; Yes = 1;	0.892	0.311

Differences between groups were assessed using t-tests for continuous variables and Chi-square tests for categorical variables, with all *p*-values < 0.05 unless otherwise noted

### Nested fixed effects model analysis

This study utilizes a nested fixed-effects model to examine the impact of physical activity, social activity, and other variables on sleep duration in middle-aged and older adults. Model I includes only marital status, the interaction term between sex and age, and the squared term for age. Model II expands on Model I by adding variables such as education level, drinking habits, and chronic diseases to account for potential confounders. Model III incorporates physical activity as the core independent variable and an interaction term to explore the interaction effects between variables.

**Fig. 3:**
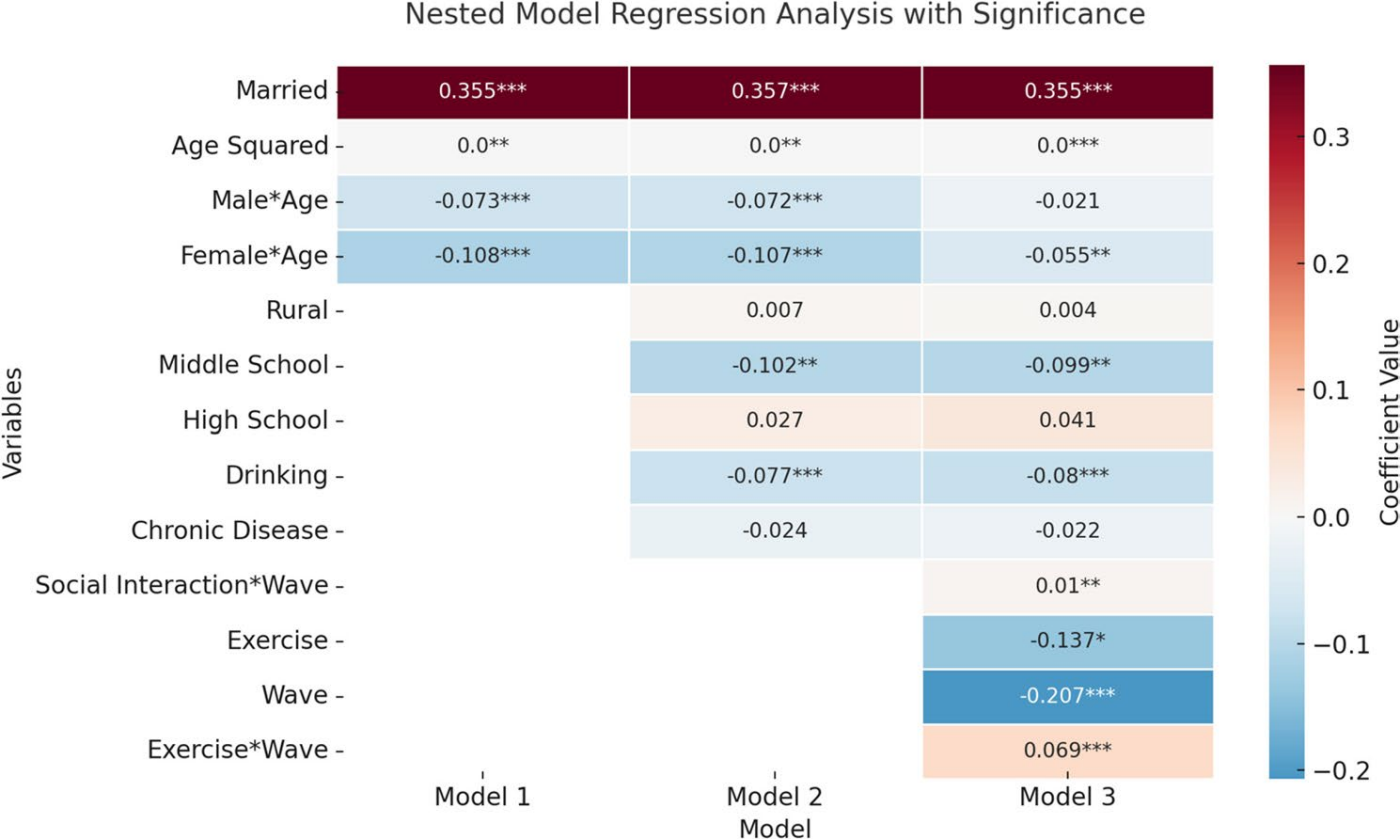
Nested fixed effects model analysis.s Analysis Heatmap. Note: *** *p* < 0.01, ** *p* < 0.05, * *p* < 0.1.

Figure 3 visually represents each variable’s coefficients, significance, and direction, providing clear visual support for interpreting the models. The color gradients in the heat map indicate the strength of each variable’s effect on sleep duration, with dark red representing a positive effect and dark blue indicating a negative effect. Significance levels are denoted by asterisks, facilitating the rapid identification of key variables.

#### Model 1: baseline demographic factors

Model 1 analyzes the effects of fundamental demographic variables on sleep duration in middle-aged and older adults. The results indicate that marital status significantly influences sleep duration (*β* = 0.355, *p* < 0.001), with married individuals sleeping longer than their unmarried counterparts. The age-squared term reveals a quadratic, non-linear relationship with a significant positive coefficient (*β* = 0.000, *p* < 0.05). The interaction term between sex and age shows a negative effect for both males (*β* = -0.073, *p* < 0.001) and females (*β *= -0.108, *p* < 0.001), suggesting that sleep duration decreases with age for both genders, with a greater reduction observed in females.

#### Model 2: incorporation of socioeconomic and health factors

In Model 2, variables such as education level, drinking habits, and chronic diseases were incorporated to examine their effects on sleep duration in middle-aged and older adults. The results indicated that middle school education significantly negatively affected sleep duration compared to primary school education (*β* = -0.102, *p* < 0.05). In contrast, the effect of high school education was insignificant (*β* = 0.027, *p* = 0.141). Drinking habits were associated with a significant reduction in sleep duration compared to non-drinkers (*β* = -0.077, *p* < 0.001), suggesting that alcohol consumption negatively influences sleep. However, the effect of chronic diseases was not statistically significant (*β* = -0.024, *p* = 0.183).

When these variables were included, the coefficient for marital status remained stable (*β* = 0.357, *p* < 0.001), indicating a consistent and significant effect of being married on sleep duration compared to being unmarried. Additionally, the coefficients for the interaction terms between gender and age slightly decreased (males: *β* = -0.072, *p* < 0.001; females: *β* = -0.107, *p* < 0.001), but they remained significant, further highlighting the importance of these interaction effects.

#### Model 3: incorporation of physical and social activity variables

Model 3 incorporates physical activity, social activity, and their interaction terms with time into Model 2. The results indicate that while the direct effect of physical activity on sleep duration is negative in the short term (*β* = -0.137, *p* = 0.019), its interaction term with time shows a significant positive effect (*β* = 0.069, *p* < 0.01). This finding underscores the core argument of this study: that the benefits of physical activity on sleep health are not instantaneous but are accrued over time, requiring sustained, long-term engagement. This suggests that for each additional survey wave (approximately 2–3 years), individuals engaging in physical activity experience an increase of approximately 4.1 min (0.069 h) in sleep duration compared to non-exercisers. Over the 9-year study period, this translates to a cumulative difference of approximately 16–20 min of additional sleep. Furthermore, the interaction term for social activity with time also significantly influences sleep duration (*β* = 0.010, *p* < 0.1), though its relatively small coefficient implies a more modest improvement. A direct comparison of the interaction coefficients (0.069 vs. 0.010) reveals that the long-term cumulative benefit from physical activity is substantially larger than that from social activities. Upon including these key behavioural variables and their temporal interactions, the negative effect of drinking habits observed in Model 2 remained highly significant with a similar magnitude (*β* = -0.080, *p* < 0.001).

The findings of this study provide valuable insights for developing health intervention policies targeted at middle-aged and elderly populations. While the interaction effect of physical activity with time (*β* = 0.069) demonstrates statistical significance for long-term benefits, it is important to note that the absolute effect size of marital status (*β* = 0.355) on sleep duration is larger. However, physical activity represents a modifiable behaviour with cumulative benefits over time, making it particularly relevant for intervention strategies, whereas marital status is less amenable to direct intervention. The nested model analysis further reveals that the adverse effect of the female-age interaction term on sleep duration remains statistically significant in Model 3 (*β* = -0.055, *p* < 0.05). However, its magnitude is reduced compared to Model 1 (*β* = -0.108, *p* < 0.001) and Model 2 (*β* = -0.107, *p* < 0.001). This suggests that the gender-specific, age-related decline in sleep duration is a robust phenomenon, but one that is partially associated with an individual’s engagement in health-promoting behaviours, as demonstrated by the attenuation of the coefficient after their inclusion. The long-term cumulative benefits of physical and social activities, as captured by their interaction with the wave variable, persist despite the overall negative age effect on sleep duration, highlighting that these two processes are independent but co-occurring. This highlights the age-related gender differences in how exercise and social activities influence sleep duration among middle-aged and older adults.

The model analysis further reveals that the effect of physical activity on sleep duration in middle-aged and older adults changes over time. Short-term physical activity reduces sleep duration, whereas long-term engagement improves it. In contrast, the direct impact of social activities on sleep duration is modest. While the interaction term with time shows a positive effect, it is considerably smaller than the effect of the interaction term of physical activity, emphasising that although social activities contribute to sleep improvement, their influence is less pronounced than that of physical activity.

The nested fixed-effects model analysis results indicate that physical activity significantly influences sleep duration among middle-aged and older adults. Moreover, physical activity had a more substantial long-term effect on sleep duration than social activities.

## Model validation

### Hausman check

To address potential endogeneity arising from unobserved individual heterogeneity (e.g., genetic predisposition to sleep issues or personality traits), we employed the Hausman specification test. The null hypothesis assumes that the coefficients of the random effects model are consistent and valid. In contrast, the alternative hypothesis posits that the coefficients of the fixed effects model are consistent, but those of the random effects model are not. The essence of the test lies in comparing the coefficients from both models. If the difference between them is statistically significant, the null hypothesis is rejected, and the fixed effects model is preferred. Conversely, if the difference is insignificant, the null hypothesis is accepted, and the random effects model is chosen [20]. The test results (χ² = 172.65, *p* < 0.001) reject the assumption of consistency for the random-effects estimator, indicating that the fixed-effects model is more suitable for the data analysis in this study. By using the fixed-effects specification, this study effectively controls for time-invariant endogenous variables that could bias the relationship between health behaviours and sleep duration.

### Robustness check

This paper tests the linear regression, random, and fixed-effects models to verify the robustness of its findings. This process does not seek identical coefficients but rather aims to confirm that the direction and statistical significance of the key variables’ effects remain consistent across different model specifications. By comparing the results of these three models, the robustness of the model can be assessed. If the results from different models are consistent, the model’s conclusions are more reliable. However, if there are significant differences in the results, the reasons should be further analysed [21]. Table 2 shows that the effects of the main variables are consistent across the models, which further enhances the reliability of the findings.

**Table 2:** Robustness Test

	Model 1 Linear regression model	Model 2 Random effects model	Model 2 Fixed effects model
Married	0.202^***^(0.027)	0.238^***^(0.028)	0.355^***^(0.053)
Female * age	-0.081^***^(0.010)	-0.879^***^(0.010)	-0.055(0.027)
Male *Age	-0.075^***^(0.010)	-0.082^***^(0.010)	-0.021(0.028)
Square of age	0.001^**^(0.000)	0.001^***^(0.000)	0.000^**^(0.000)
Population	0.040^*^(0.019)	0.020(0.024)	0.004(0.053)
Junior high school	0.067^***^(0.019)	0.038(0.025)	-0.100^*^(0.050)
Senior high school and above	0.128^***^(0.023)	0.106^***^(0.033)	0.041(0.087)
Whether or not you drink alcohol	-0.102^***^(0.018)	-0.095^***^(0.019)	-0.081^**^(0.027)
Presence of chronic diseases	-0.384^***^(0.018)	-0.273^***^(0.021)	-0.022(0.032)
Attendance at social events * Wave	0.013^**^(0.004)	0.010^**^(0.004)	0.010^*^(0.005)
Engage in activity	-0.072(0.079)	-0.102(0.066)	-0.137(0.075)
Wave	-0.112^***^(0.020)	-0.129^***^(0.016)	-0.207^**^(0.049)
Engage in exercise * Wave	0.049^*^(0.021)	0.060^***^(0.017)	0.069^***^(0.019)
Constant term	9.292^***^(0.315)	9.476^***^(0.309)	7.307^***^(1.229)
R-squared	0.031^***^	0.030^***^	-0.630^***^

* *p* < 0.05, ** *p* < 0.01, *** *p* < 0.001

### Heterogeneity analysis

Education level groups conducted the heterogeneity analysis for several theoretical and methodological reasons. First, education level serves as a proxy for socioeconomic status and health literacy, both of which significantly influence the adoption of health behaviours and sleep hygiene practices. Second, educational attainment affects individuals’ access to health information and their ability to implement lifestyle modifications effectively. Third, previous literature suggests that the relationship between physical activity and sleep outcomes varies across different socioeconomic strata. Education level was selected over other demographic factors (such as income or occupation) because it remains relatively stable throughout adulthood and is less susceptible to reverse causation bias in longitudinal studies.

**Fig. 4:**
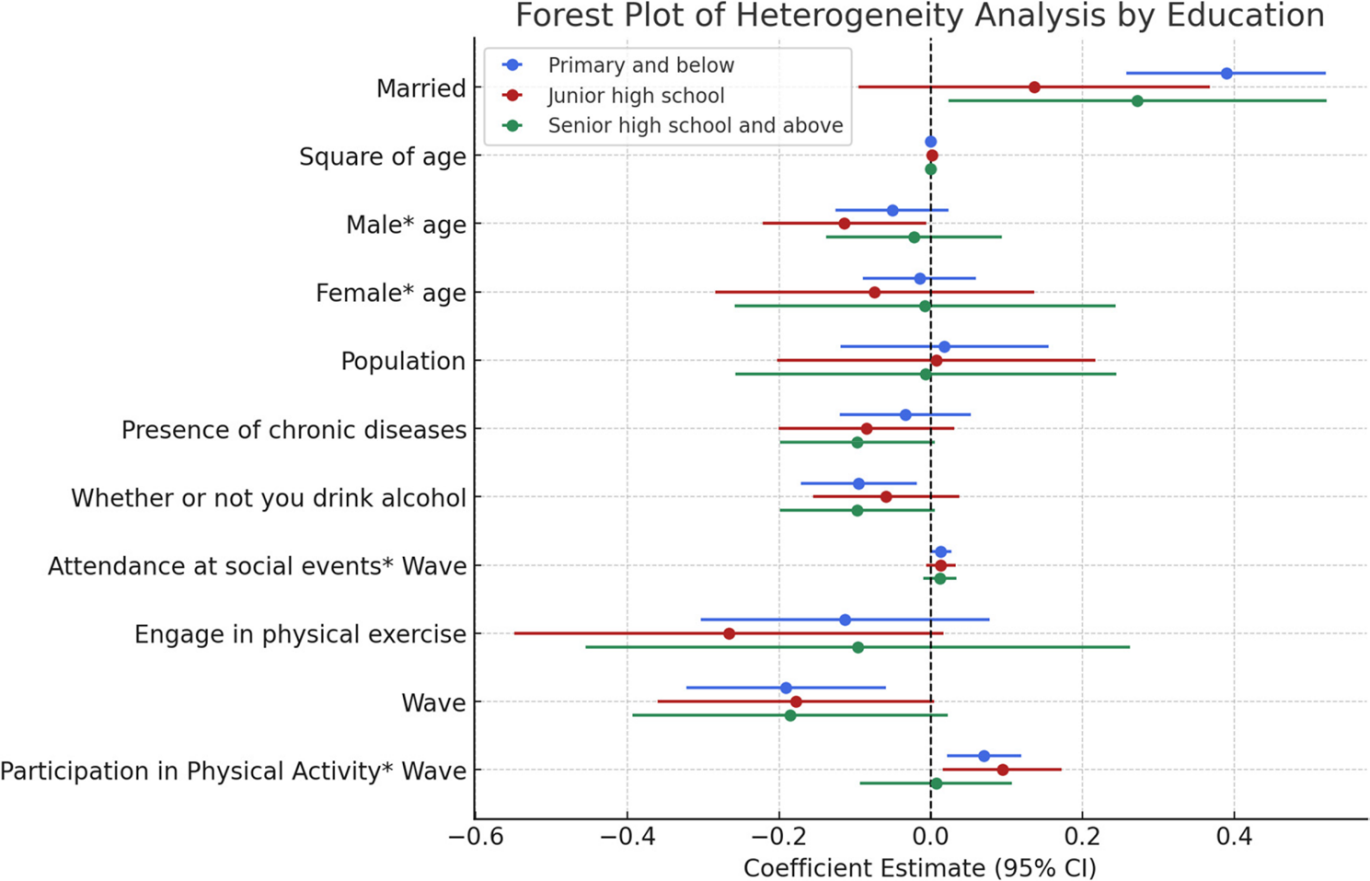
Forest Plot of Heterogeneity Analysis by Education

In this study, heterogeneity analyses were conducted to examine differences in factors influencing sleep duration among middle-aged and older adults from different educational background groups. Please refer to Fig. 4 for further details.

In the primary school and below education level group, the effect of marital status on sleep duration was significantly positive (*β* = 0.389, *p* < 0.001). In contrast, in the junior high school education level group, the effect was not significant (*β* = 0.136, *p* > 0.1). In the high school and above education level group, marital status had a significantly positive impact (*β *= 0.272, *p* < 0.05).

The squared term of age was significantly positive in both the primary school and below group (β = 0.000, *p* < 0.10) and the junior high school group (*β* = 0.001, *p* < 0.05). However, the squared age term coefficient was insignificant in the high school and above group (*β* = 0.000, *p* > 0.10). The interaction term between males and age was significantly negative in the lower secondary education level group (*β* = -0.114, *p* < 0.05), but not significant in the primary school and below (*β* = -0.051, *p* > 0.1) and high school and above groups (*β* = -0.022, *p* > 0.1). The interaction term between females and age was not significant across the three education groups.

Regarding chronic disease, the coefficient was significantly negative only in the high school and above education level group (*β* = -0.097, *p* < 0.10), with no significant effect in the junior high school or primary school and below groups.

Drinking behaviour had a significantly negative effect (*β* = -0.095, *p* < 0.05) in the primary school and below group, whereas alcohol consumption had a non-significant effect in the junior high school and high school and above groups.

For physical activity, the direct effect was negative in the primary school and below group (*β* = -0.113, *p* > 0.1) and significantly negative in the middle school group (*β* = -0.266, *p* < 0.10). The interaction between physical activity and time was significantly positive in the middle school group (*β* = 0.094, *p* < 0.05). In contrast, neither the direct effect nor the interaction term for physical activity was significant in the high school and above group.

Heterogeneity analyses underscore the significant moderating role of education level in the factors affecting sleep duration in middle-aged and older adults. The findings from the heterogeneity analysis reveal that the long-term cumulative benefits of physical activity on sleep duration are most pronounced in the middle school education group. The effect of social activities and sleep duration is significant only in the primary school and below group. These results underscore the significant moderating role of education level in how these behaviours affect sleep over time.

## Discussion

### Temporal effects of exercise: physical activity on sleep duration

Interestingly, the data revealed a crucial temporal dynamic for physical activity, showing that while a short-term, immediate association with sleep duration might be negative, the long-term, cumulative engagement has a robust positive effect. This pattern strongly suggests that the full realisation of sleep benefits from physical activity is not instantaneous but necessitates long-term adherence. Consistent and sustained physical activity gradually enhances sleep duration by regulating physiological rhythms, effectively alleviating psychological stress, and improving overall physical function [22]. This finding provides a nuanced perspective beyond cross-sectional studies by demonstrating the ‘maturation’ of the protective effect of exercise over time, a mechanism previously linked to better health and overall well-being in older adults [23].

### Impact of social activity on sleep duration

Notably, a positive, though comparatively more modest, long-term effect of social activity on sleep duration was also confirmed in our longitudinal analysis. Active participation in social activities is theorised to provide crucial emotional support, effectively mitigating feelings of loneliness and, indirectly, improving sleep outcomes by enhancing mental health [24]. Specifically, social engagement can improve both the subjective experience and objective quality of sleep by decreasing loneliness and depressive symptoms, particularly among older adult individuals living alone or widowed [25]. Furthermore, social activity may indirectly enhance sleep duration by promoting cognitive function and fostering psychological resilience [26]. This cumulative benefit, although smaller than that observed for physical activity, reinforces the idea that over time, the psychosocial benefits of social integration progressively strengthen their positive influence on sleep health.

### Differential mechanisms of exercise: physical activity and social activity

**Fig. 5:**
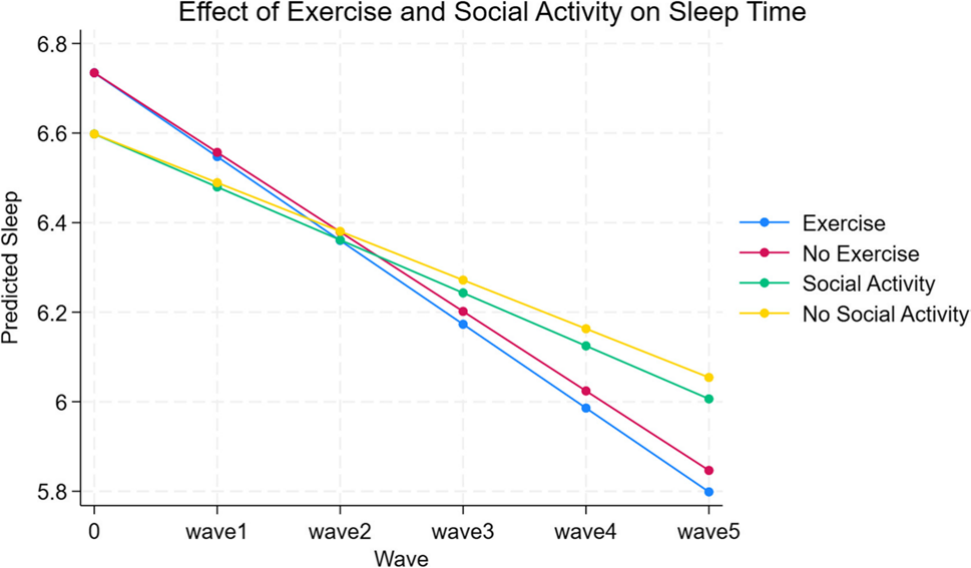
Interaction Effect Diagram, Note: wave1: first wave survey; wave2: second wave survey; wave3: third wave survey; wave4: fourth wave survey

Surprisingly, our findings suggest distinct temporal patterns that align with theoretical physiological and psychosocial pathways, although specific biological mechanisms were not directly tested in this model.ageingactivity appears to exert a more direct and substantial long-term effect, primarily through physiological mechanisms, such as optimising circadian rhythms, enhancing cardiovascular function, and regulating neuroendocrine markers. In contrast, social activity contributes to sleep improvement indirectly, predominantly via psychosocial mechanisms, in which sustained engagement reduces feelings of loneliness and alleviates depressive symptoms, thereby fostering emotional support and overall mental well-being [27, 28]. This novel separation of long-term mechanisms reinforces the theoretical framework proposed in Fig. 5, providing empirical evidence for the ‘Cumulative Bridge’ concept, where sustained behavioural inputs lead to greater long-term sleep benefits.

Notably, our heterogeneity analysis revealed a significant moderating effect of educational attainment on the long-term impact of health behaviours on sleep duration. Specifically, the most pronounced long-term cumulative benefits of physical activity were observed among the middle school education group, whereas the positive temporal effect of social activities was evident only in the primary school and below group. This differential impact suggests that educational level serves as a critical proxy for health literacy and socioeconomic status, influencing both the adoption of and the physiological response to lifestyle modifications [29–30]. Individuals with lower educational attainment may have less access to comprehensive health information or structured exercise facilities, making the adoption of regular physical activity a greater health gain [31–32], which subsequently translates into a stronger temporal effect on sleep. These findings resonate with the ‘Fundamental Cause Theory’ of health disparities, which posits that individuals with higher socioeconomic status (proxied here by education) are better positioned to deploy flexible resources—such as health literacy and agency—to maximise the health returns of behavioural interventions. The pronounced effect in the educated groups suggests that education may enhance the ‘efficiency’ of translating physical activity into sleep benefits through better adherence and sleep hygiene practices. Prior research also highlights that health literacy—closely linked to educational level—is a determinant of adopting and maintaining healthy behaviours, including sleep-related practices [33–34]. Moreover, meta-analytic evidence suggests that socioeconomic status moderates the conversion of behavioural intentions into actual physical activity [35]. From a theoretical perspective, the fundamental cause theory underscores that education, as a key component of socioeconomic status, persistently shapes health disparities by providing or limiting access to flexible resources such as knowledge, social support, and power [36]. This is a crucial novel finding that directly informs policy interventions, underscoring the necessity of designing targeted, educationally sensitive public health programs to maximise the sleep benefits for the most vulnerable segments of the ageing population.

Furthermore, the visualisation in Fig. 5 dynamically illustrates the ‘Cumulative Bridge’ concept, with the temporal trends providing a clear interpretation of the short-term adverse and long-term positive effects of physical activity on predicted sleep duration. Specifically, in the initial waves, the predicted sleep duration for the exercise group (blue line) declines more steeply than for the non-exercise group (red line), which is consistent with the estimated immediate adverse direct effect of exercise. However, over subsequent follow-up periods, this trajectory reverses: the exercise curve’s decline substantially slows, while the non-exercise curve continues its descent, leading to a gradually widening gap in favour of the sustained exercise group in later waves. This dynamic shift clearly demonstrates how the long-term engagement effect effectively mitigates age-related declines in sleep duration, supporting the idea that the protective effect of physical activity on sleep duration increases cumulatively over time by improving cardiovascular function, regulating melatonin secretion, and reducing cortisol levels [37]. In contrast, the impact of social activity on sleep duration suggests a positive effect that emerges earlier, as it helps alleviate loneliness and depressive symptoms, provides emotional support, and enables middle-aged and older adults to experience better sleep early on [38]. Unlike activity, improving sleep through social activity primarily occurs through psychological pathways, with limited direct effects on physiological mechanisms [39]. Nevertheless, the findings highlight that activity and social activity act as complementary components in health interventions [40]. exercise offers long-term physiological benefits, while social activity can effectively alleviate stress and loneliness in the short term [41].

### Effects of marital status, drinking behaviour, and gender differences

The fixed-effects model robustly confirmed that marital status provides a substantial protective effect on sleep duration in middle-aged and older adults. Marital relationships are believed to indirectly enhance sleep duration by reducing stress perceptions, providing consistent emotional support, and promoting adherence to healthy behaviours [42]. Moreover, the quality of the marriage relationship critically affects sleep outcomes: high-quality relationships are associated with reduced sleep disorders, while marital conflict can negatively affect sleep [43]. Conversely, the present study found a consistent and significant negative association between drinking behaviour and sleep duration, suggesting that chronic alcohol consumption is a critical, yet modifiable, factor contributing to the reduction of sleep quality and duration in this demographic. Although alcohol may initially aid sleep onset, the consensus is that it disrupts sleep architecture, leading to fragmented sleep and early awakening problems [44–45]. This negative influence is further compounded as chronic consumption may increase the risk of insomnia and sleep disorders by affecting the balance of neurotransmitters [46]. Finally, the interaction effect of sex and age showed a greater age-related decline in sleep duration in women. This gender-specific acceleration may be attributed to hormonal fluctuations, particularly during the menopausal stage in women, which can substantially increase the prevalence of sleep disorders [47].

### Limitations

This study has several important limitations that should be considered when interpreting the findings. First, sleep duration was measured via self-reported questionnaires, which may introduce recall and social desirability biases. Objective sleep measurement devices could provide more accurate assessments in future studies. Second, the study experienced attrition across the nine-year follow-up period, which may introduce selection bias if dropout was related to sleep patterns or health behaviours. Third, the generalizability of findings may be limited to the Chinese cultural context, and cross-cultural validation would strengthen the evidence base. Fourth, although we controlled for several important confounders, unmeasured variables, such as specific mental health conditions, detailed medication use, sleep disorders, and environmental factors, may influence the observed relationships. Fifth, the discrete-time specification, while interpretable, may not capture more nuanced temporal dynamics that could be revealed by continuous-time modelling. Finally, the study measures sleep duration rather than comprehensive sleep quality, which includes factors such as sleep efficiency, sleep latency, and subjective sleep satisfaction. Furthermore, our reliance on a binary exposure measure precludes the examination of intensity-specific dose-response relationships. Additionally, while we propose physiological and psychosocial mechanisms to explain the observed associations, we lacked the specific biomarkers or intermediate psychological variables to conduct formal mediation analyses, making these mechanistic interpretations theoretical rather than empirical.

We also acknowledge the potential for bidirectional relationships, or reverse causality, where individuals with superior sleep health may possess the energy reserves necessary to initiate and sustain physical activity. While our fixed-effects strategy controls for time-invariant individual heterogeneity, dynamic selection bias remains a possibility. However, the observed gradual widening of the sleep duration gap over time supports the hypothesis of a cumulative protective effect of activity rather than a selection effect in which good sleepers simply start exercising.

## Conclusions

This study finds that long-term participation in and social activities positively affects sleep duration, with physical exercise showing a more pronounced long-term impact. Marital status significantly protects sleep duration, while alcohol consumption has a notable negative effect on sleep time. The non-linear relationship between age and sleep duration, along with gender differences, further underscores the complexity of sleep issues in middle-aged and older adults. Building on existing research, this study further validates the multi-dimensional impact of health behaviours on sleep duration, emphasising the complementary role of physical exercise, physical activity, and social activities.

The findings suggest specific policy directions: (1) Public health programs should emphasize sustained, long-term physical activity engagement rather than short-term intensive interventions, given the temporal dynamics observed; (2) Community-based social activity programs should be integrated with physical activity initiatives to maximize complementary benefits; (3) Health education should address alcohol consumption patterns as part of comprehensive sleep hygiene promotion; (4) Interventions should consider the differential effects across educational levels, with particular attention to lower-educated populations who showed stronger responses to behavioral interventions.

## Data Availability

The datasets generated and/or analyzed during the current study are available in the China Health and Retirement Longitudinal Study (CHARLS) repository, which is publicly accessible. Data access can be requested through the official CHARLS website at [http://charls.pku.edu.cn/en/](https://www.google.com/url?sa=E&q=http%3A%2F%2Fcharls.pku.edu.cn%2Fen%2F).
